# Dietary Advanced Glycation Endproducts Decrease Glucocorticoid Sensitivity In Vitro

**DOI:** 10.3390/nu12020441

**Published:** 2020-02-10

**Authors:** Timme van der Lugt, Antje R. Weseler, Misha F. Vrolijk, Antoon Opperhuizen, Aalt Bast

**Affiliations:** 1 Department of Pharmacology and Toxicology, Faculty of Health, Medicine, and Life Sciences, Maastricht University, 6200 MD Maastricht, The Netherlands; a.weseler@maastrichtuniversity.nl (A.R.W.); m.vrolijk@maastrichtuniversity.nl (M.F.V.); antoon.opperhuizen@maastrichtuniversity.nl (A.O.); a.bast@maastrichtuniversity.nl (A.B.); 2 Faculty of Science and Engineering, Campus Venlo, Maastricht University, 5911 AA Venlo, The Netherlands; 3 Office for Risk Assessment and Research (BuRO), Dutch Food and Consumer Safety Authority (NVWA), 3540 AA Utrecht, The Netherlands

**Keywords:** dietary advanced glycation endproducts, glucocorticoid resistance, inflammatory bowel diseases, inflammation, reactive oxygen species

## Abstract

Glucocorticoids are very effective anti-inflammatory drugs and widely used for inflammatory bowel disease (IBD) patients. However, approximately 20% of IBD patients do not respond to glucocorticoids and the reason for this is largely unknown. Dietary advanced glycation endproducts (AGEs) are formed via the Maillard reaction during the thermal processing of food products and can induce a pro-inflammatory reaction in human cells. To investigate whether this pro-inflammatory response could be mitigated by glucocorticoids, human macrophage-like cells were exposed to both LPS and AGEs to induce interleukin-8 (IL8) secretion. This pro-inflammatory response was then modulated by adding pharmacological compounds interfering in different steps of the anti-inflammatory mechanism of glucocorticoids: rapamycin, quercetin, and theophylline. Additionally, intracellular reactive oxygen species (ROS) were measured and the glucocorticoid receptor phosphorylation state was assessed. The results show that AGEs induced glucocorticoid resistance, which could be mitigated by quercetin and rapamycin. No change in the phosphorylation state of the glucocorticoid receptor was observed. Additionally, intracellular ROS formation was induced by AGEs, which was mitigated by quercetin. This suggests that AGE-induced ROS is an underlying mechanism to AGE-induced glucocorticoid resistance. This study shows for the first time the phenomenon of dietary AGE-induced glucocorticoid resistance due to the formation of ROS. Our findings indicate that food products with a high inflammatory potential can induce glucocorticoid resistance; these results may be of great importance to IBD patients suffering from glucocorticoid resistance.

## 1. Introduction

The use of glucocorticoids is increasing due to the increased prevalence of chronic inflammatory diseases, such as asthma, rheumatoid arthritis, and inflammatory bowel diseases (IBD). Despite the effectiveness of glucocorticoids as anti-inflammatory drugs, some patients do not experience the anticipated therapeutic response and appear to be resistant to these drugs [1,2,3]. The underlying molecular mechanisms of such glucocorticoid resistance is still poorly understood.

Under physiological circumstances, glucocorticoids diffuse across the cell membrane and bind to the alpha form of the glucocorticoid receptor (GR) [4]. After binding, the GR–glucocorticoid complex translocates to the nucleus and exerts its anti-inflammatory effect in several ways. Within the nucleus, GR homodimerizes and binds to glucocorticoid responsive elements (GRE) in the promotor region of glucocorticoid responsive genes, thereby switching on anti-inflammatory genes [5]. These genes include genes encoding for β2 adrenergic receptors and mitogen-activated protein (MAP) kinase phosphatase-1 (MKP-1), which inhibits MAP kinases and thereby switches off pro-inflammatory gene expression [6,7]. In addition, activated GR can increase histone deacetylation by recruiting histone deacetylase 2 (HDAC2), leading to a reduced expression of pro-inflammatory genes activated by the nuclear factor kappa-light-chain-enhancer of activated B cells (NF-κB). Lastly, glucocorticoids can also directly enhance the degradation of the mRNA of pro-inflammatory genes, such as that of tumor necrosis factor (TNF)-alpha [8,9].

Although the anti-inflammatory mechanisms of the action of glucocorticoids are well understood, it is still unknown why glucocorticoid resistance occurs in some patients. The molecular mechanisms by which glucocorticoid resistance could arise have been reviewed by Barnes [3]. Phosphorylation of the GR seems to play an important role in causing glucocorticoid resistance. After phosphorylation of the receptor, its stability and its interaction with other proteins are altered, and nuclear translocation is decreased, thereby inhibiting its ability to bind to GRE [10,11,12]. Two specific proposed phosphorylation sites of the GR are serine (Ser) 226 and 211 [13,14]. These sites can be phosphorylated in several ways. For instance, the mammalian target of rapamycin (mTOR) activates C-Jun N-terminal kinase (JNK), which directly phosphorylates Ser 226 of the GR. Additionally, the GR can be phosphorylated through the activation of p38 mitogen-activated protein kinases (p38MAPK) [14]. Compounds inducing cell stress, such as reactive oxygen species (ROS), can lead to an increase in phosphoinositide 3-kinase delta (pI3Kδ) and mTOR complex 1 activity, which eventually lead to less GR nuclear translocation through phosphorylation.

Furthermore, ROS can damage and thereby inactivate HDAC2 which contributes to a reduced sensitivity to glucocorticoids [15,16]. Hence, antioxidants, such as the polyphenol quercetin, could counteract the effects of ROS, ameliorating the sensitivity to the anti-inflammatory effects of glucocorticoids [17].

Recently, a study of ours was one of the first to show that dietary advanced glycation endproducts (AGEs) cause inflammation in human macrophages in vitro by binding to the receptor for advanced glycation endproducts (RAGE) [18]. After activation, RAGE signaling leads to the activation of NF-κB via PI3K and additionally to the activation of p38MAPK. Furthermore, AGEs increase the formation of intracellular ROS [19,20]. Therefore, we hypothesized that dietary AGEs may induce glucocorticoid resistance in human cells of the innate immune response.

Therefore, the present study was aimed at investigating the effects of dietary AGEs on the anti-inflammatory effects of cortisol in human macrophages and to unravel the underlying molecular mechanisms by which dietary AGEs induce glucocorticoid resistance in these cells.

## 2. Materials and Methods

### 2.1. Chemicals and Reagents

Casein from bovine milk, α-lactose monohydrate, NaOH, sodium phosphate, cortisol, theophylline, rapamycin, quercetin dihydrate, 2′,7′-dichlorofluorescin diacetate (H2DCF-DA), 2-mercaptoethanol, and thiazolyl blue tetrazolium bromide (MTT) were obtained from Sigma-Aldrich (Saint Louis, MO, USA). D-glucose, glutamate, fetal bovine serum (FBS), and Dulbeccco’s Phosphate-Buffered Saline (DPBS) were obtained from Gibco (Thermo Scientific, Waltham, MA, USA). Sodium chloride, Triton-X, Tris base, LPS, and phorbol 12-myristate 13-acetate (PMA) were obtained from Sigma (Zwijndrecht, Netherlands).

### 2.2. Preparation of Dietary AGEs

Dietary AGEs were prepared as described previously [18]. In short, casein, glucose, and lactose, in the proportions of milk powder (11 mM glucose, 0.2 M lactose, 10 g/L casein from bovine milk), were dissolved in 50 mM phosphate buffer (pH = 7.4) and heated in an Erlenmeyer flask on a heating plate at 100 °C for up to 15 min. After 15 min, a sample was taken out and put in ice water. Samples were aliquoted and stored at −80°C. The concentrations of several individual AGEs have been published in our previous study [18]. MG-H1 was present in concentrations of 1.6 ± 0.9 μg/mL (mean ± standard deviation (SD)); the CML concentration was 0.6 ± 0.5 μg/mL.

### 2.3. Determination of Endotoxin in Dietary AGEs

Endotoxin presence in the AGE preparation was assessed by the PYROGENT Gel Clot LAL Assay with a 0.06 EU/mL sensitivity (Lonza, Basel, Switzerland). The assay was performed in accordance to the manufacturer’s protocol using endotoxin-free dilutions and reagent tubes (Lonza, Basel, Switzerland). Only endotoxin-free samples were used in the cell culture experiments.

### 2.4. Cell Culture and Exposure

THP-1 monocytes (ATCC, TIB-202) cultured in RPMI 1640 with L-glutamine, Hepes, and phenol red (Gibco, Thermo Scientific, Waltham, MA, USA) supplemented with 10% (v/v) FBS, D-glucose (4.5 g/L), Na-pyruvate (1 mM), and 2-mercaptoethanol (50 μM) were seeded in a 96-well plate in a cell density of 70,000 cells/well and differentiated into macrophages by adding 200 nM of PMA to the cell culture medium and culturing them for 72 h. After differentiation, the cells were exposed to 10% (v/v) AGEs, different concentrations of LPS, cortisol, theophylline, rapamycin, and quercetin in serum and phenol red free culturing medium and always in a 50% DPBS solution. The exposure concentrations of the compounds in the AGEs were 0.16 ± 0.09 μg/mL MG-H1, 0.06 ± 0.05 μg/mL CML, 1.1 mM glucose, 20 mM lactose, and 1 g/L casein. The control conditions included phenol red free culturing medium and 50% DPBS. Ethanol (maximum 0.17 v/v%) was used as the vehicle control for cortisol, theophylline, and quercetin. DMSO (0.1 v/v%) was used as the vehicle control for rapamycin.

### 2.5. Quantification of Interleukin-8 (IL8) Release by ELISA

Interleukin-8 (IL8) release in cell culture supernatant was assessed by a commercially available ELISA kit (R&D systems, Minneapolis, MN, USA). The assay was performed in accordance with the manufacturer’s protocol.

### 2.6. Quantification of Glucocorticoid Receptor Protein Levels by Western Blot

THP-1 monocytes were seeded in a 6-well plate with a cell density of 2,100,000 cells/mL and differentiated into macrophages by adding 200 nM of PMA to the cell culture medium and culturing them for 72 h. After differentiation, cells were exposed to 50% DPBS, 10% (v/v) AGEs, and 3 ng/mL LPS for 24 h. After incubation, cells were lysed with a Triton buffer consisting of 150 mM sodium chloride, 0.1% Triton-X, and 50 mM Tris pH 8, and the protein content was assessed with the BCA Assay (Thermo Scientific, Waltham, USA) according to the manufacturer’s protocol.

A sample of 10 µg with reducing laemmli buffer was loaded onto a 10% mini protean TGX precast gel (Bio-rad, Hercules, CA, Verenigde Staten) and run in a Bio-rad cell. Protein was transferred to an Immobilon-P PVDF Membrane (Merck, Darmstadt, Germany). Membranes were analyzed using an Amersham Imager 600 (GE Healthcare, Chicago, IL, USA). The primary antibodies used were Glucocorticoid Receptor (D6H2L), Phospho-Glucocorticoid Receptor (Ser211), and β-actin (13E5) from Cell Signaling Technology (Danvers, MA, USA) and Anti-phospho-Glucocorticoid receptor Ser226 from Millipore (Temecula, USA). The secondary antibody used was Anti-rabbit IgG, HRP-linked Antibody (Cell Signaling Technology).

### 2.7. Quantification of ROS Levels by DCFH-DA Assay

Intracellular ROS levels were quantified using the DCFH-DA assay. This assay measures intracellular ROS by their capacity to oxidize 2′,7′-dichlorofluorescin diacetate (H2DCF-DA) to the fluorescent DCF [21]. In this study, THP-1 monocytes were cultured and seeded into a 96-well black plate/clear bottom with PMA according to the aforementioned method. After differentiation, cells were exposed to 50 µM of H2DCF-DA for 1 h. Supernatant was removed and cells were exposed to 10% AGEs, 3 nM cortisol, and different concentrations of quercetin for 24 h. Control samples were exposed to 50% DPBS, and the vehicle control of cortisol and quercetin was ethanol. After 24 h, the fluorescence of DCF was measured at λ excitation (485 nm) and λ emission (525 nm), corrected for baseline fluorescence, and taken as a measurement for intracellular ROS. To calculate the relative values, the fluorescence of cells exposed to only regular THP-1 culture medium was taken as 100%.

### 2.8. Statistics

Experiments were performed in duplicate on different days using the average of the duplicates as one value. Data were analyzed using GraphPad Prism software (v. 5.00, GraphPad Software, San Diego, CA, USA). Western blot results were analyzed using ImageJ (v1.52e, Wayne Rasband, International Institutes of Health, USA). Data obtained were tested for normality by using the D’Agostino and Pearson omnibus normality test. Data were analyzed with the nonparametric one-way ANOVA Kruskal–Wallis test followed by Dunn’s Multiple Comparison test or the Mann–Whitney *U* test to compare two sets of treatment. The significance level was set to *p* < 0.05. The significance is indicated as * *p* < 0.05, ** *p* < 0.01, and *** *p* < 0.001.

## 3. Results

### 3.1. AGE-Induced Glucocorticoid Resistance

**Figure 1:**
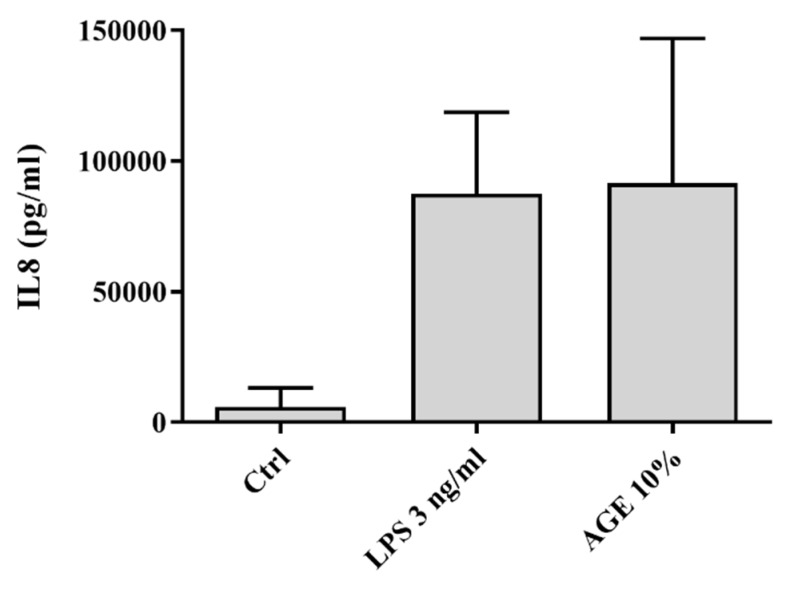
Absolute IL8 secretion of human macrophage-like cells after 24 h exposure to 3 ng/mL LPS, advanced glycation endproducts (AGEs, 10% v/v), or the control (Ctrl) condition. Data presented as mean ± SD, *n* = 3, *p* = 0.05 (Ctrl vs. LPS and Ctrl vs. AGE).

**Figure 2:**
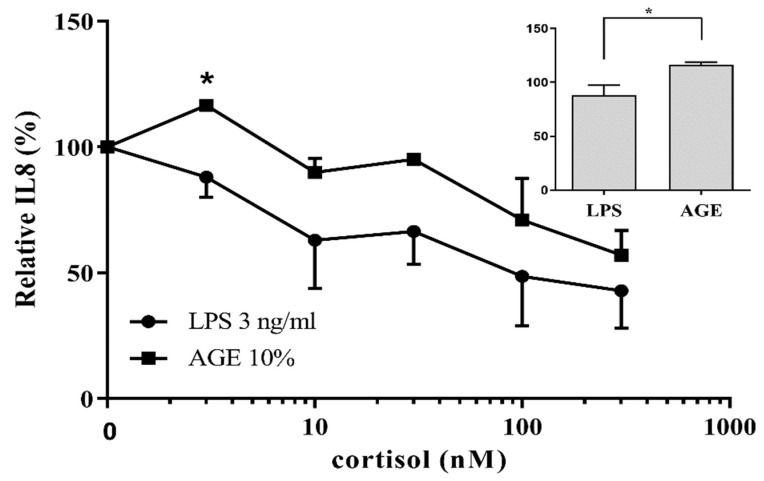
Interleukin-8 (IL8) secretion of human macrophage-like cells normalized to no cortisol after 24 h exposure to 3 ng/mL LPS or AGEs (10% v/v) and 3–300 nM of cortisol (*n* = 4). The absolute IL8 values of cells exposed to only LPS were 110 ± 20 ng/mL and to only AGEs were 154 ± 20 ng/mL. Insert: IL8 secretion of human macrophage-like cells relative to no cortisol after 24 h exposure to 3 ng/mL LPS or AGEs (10% v/v) in the presence of 3 nM cortisol (*n* = 4). Data presented as mean ± SD, *n* = 4, * *p* < 0.05 for LPS (3 ng/mL) vs. AGE (10%) in the presence of 3 nM cortisol. Statistical test used: Mann–Whitney *U*.

**Figure 3:**
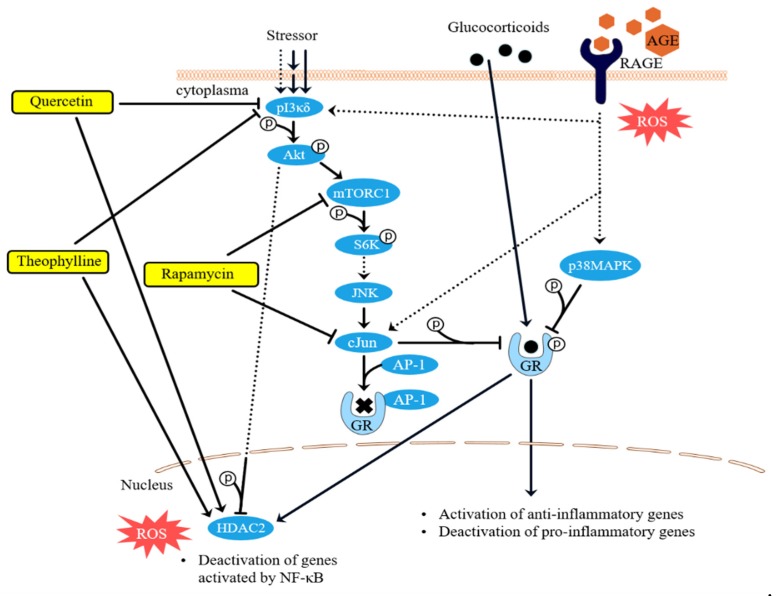
Mechanism of glucocorticoid resistance and the addition of the receptor for advanced glycation endproducts (RAGE) [3,19]. Glucocorticoids diffuse through the cell membrane into the cell where they bind to the glucocorticoid receptor (GR). This complex then translocates into the nucleus where it exerts its effect. Under glucocorticoid resistance conditions, a stressor activates phosphoinositide 3-kinase delta (pI3Kδ), thereby activating the PI3K/AKT/mammalian target of rapamycin (mTOR) pathway leading to an increase in C-Jun protein which phosphorylates the GR and induces binding of the AP-1 protein to the GR, inhibiting the glucocorticoid–GR complex from translocating to the nucleus. We suggest that AGEs bind to RAGE, thus activating both PI3K- and p38 mitogen-activated protein kinase (p38MAPK)-mediated phosphorylation of the GR. On the left, the proposed effects of different compounds (in yellow) on the glucocorticoid resistance are shown. Quercetin inhibits PI3K and protects histone deacetylase 2 (HDAC2). Rapamycin is a mTOR and a C-Jun inhibitor. Theophylline inhibits PI3K and activates HDAC.

**Figure 4:**
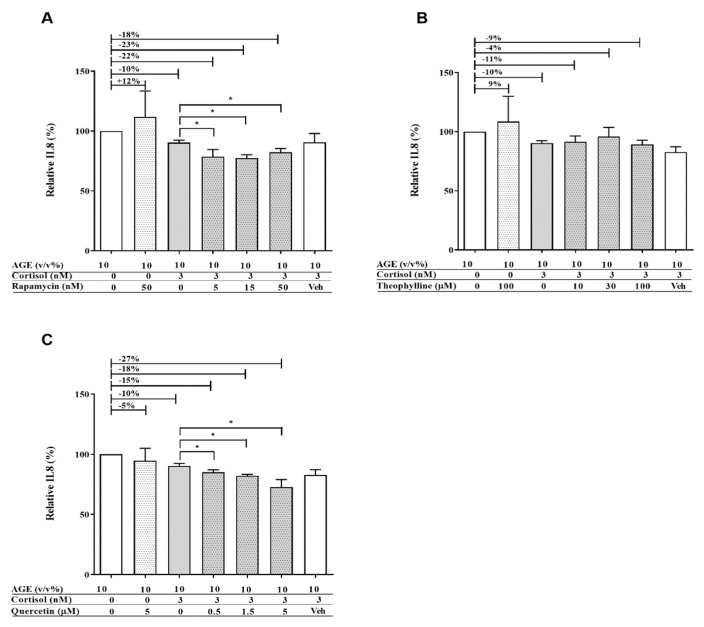
Relative IL8 secretion of human macrophage-like cells after 24 h exposure to AGEs (10% v/v), 3 nM of cortisol, and (**A**) different concentrations of rapamycin; (**B**) different concentrations of theophylline; (**C**) different concentrations of quercetin. Data presented as mean ± SD, *n* = 3, * *p* < 0.05. Statistical test used: Mann–Whitney *U*.

Differentiated THP-1 cells were exposed to different concentrations of LPS and AGEs for 24 h, after which IL8 secretion was assessed by ELISA and cell viability was assessed by MTT. No cell death occurred after exposure to LPS and AGEs (data not shown). Cells were exposed to different concentrations of LPS and AGEs (data not shown) to test which concentrations of the different compounds lead to a similar IL8 secretion. Figure 1 shows that exposing the macrophage-like cells to either 3 ng/mL LPS or 10% AGEs as eventual chosen conditions led to the same IL8 increase. To determine the anti-inflammatory effect of corticosteroids on both AGE- and LPS-induced inflammation, cells were exposed in an additional experiment to different concentrations of cortisol in combination with 10% AGEs or 3 ng/mL LPS. As shown in Figure 2, 3 nM cortisol was significantly less able to decrease the AGE-induced IL8 secretion compared to the LPS-induced IL8 secretion, which is highlighted in the insert of Figure 2. To distinguish the mechanism by which AGEs induce glucocorticoid resistance, the effect was modulated with compounds that interfered in different pathways involved in glucocorticoid resistance, as depicted in Figure 3. This further inquest into the underlying mechanism was only performed with cells exposed to AGEs. The cells were exposed to all three compounds in different concentrations with and without AGEs and cortisol. Adding different concentrations of rapamycin to human macrophage-like cells led to a significant improved cortisol response of approximately 10% (Figure 4A). However, this improvement by rapamycin did not follow a dose-dependent pattern. Theophylline alone did not have any anti-inflammatory effect and did not improve glucocorticoid responsiveness (Figure 4B). Figure 4C shows that the antioxidant quercetin itself at 5 μM did not reduce IL8 secretion, but when quercetin was added together with cortisol, IL8 secretion was reduced dose-dependently and significantly. Adding 5 µM quercetin by itself led to a decrease of only 5% (not significant), whereas the combination of 5 µM quercetin with cortisol led to a 27% reduction.

### 3.2. Phosphorylation of the Glucocorticoid Receptor

**Figure 5:**
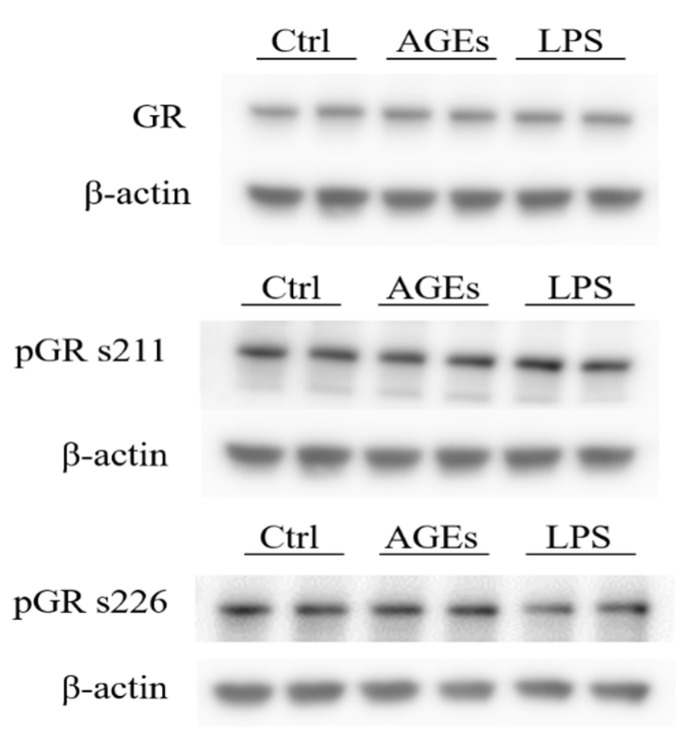
Protein levels of glucocorticoid receptor (GR), phosphorylated GR at serine (s) 211, and phosphorylated GR at serine 226. β-actin is shown as the loading control. Statistical test used: Mann–Whitney *U*.

Activation of mTOR can lead to GR phosphorylation and since the mTOR inhibitor rapamycin was able to reduce IL8 secretion compared to cortisol, the next step was to explore a possible role for GR phosphorylation in AGE-induced glucocorticoid resistance. The cells were exposed to either the control condition, 10% AGEs, or 3 ng/mL LPS. The protein levels of GR and phosphorylated GR were assessed by Western blot and calculated relatively to β-actin (Figure 5). No significant difference was found in the phosphorylation state between the different conditions.

### 3.3. Intracellular ROS Levels

**Figure 6:**
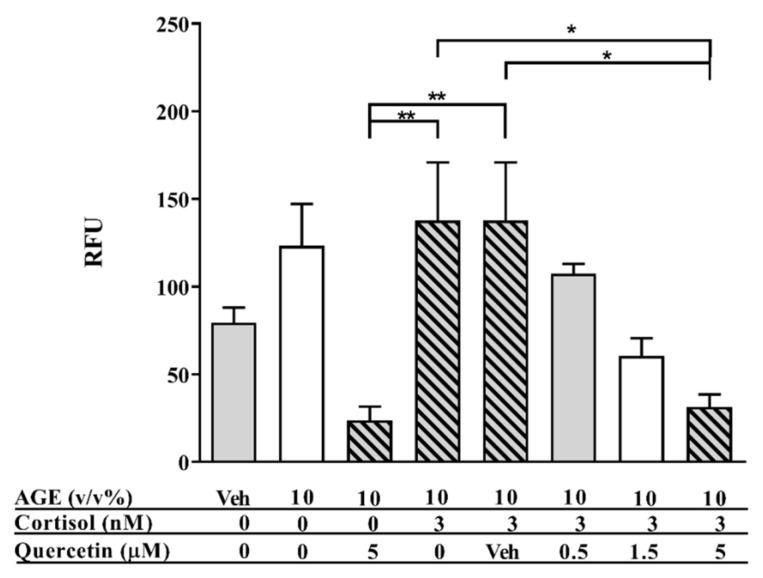
Intracellular reactive oxygen species (ROS) levels of human macrophage-like cells exposed to 10% AGEs, 3 nM cortisol (C), and different concentrations of quercetin. Baseline fluorescence of THP-1 cells was set at 100%. Data presented as mean ± SD, *n* = 4. * *p* < 0.05, ** *p* < 0.01. Statistical test used: one-way ANOVA Kruskal–Wallis.

As shown in Figure 3, quercetin dose-dependently improved glucocorticoid responsiveness. To determine whether this could be related to the mitigation of intracellular ROS levels by quercetin, cells were exposed to 10% AGEs, 3 nM cortisol, and different concentrations of quercetin for 24 h, after which intracellular ROS levels were measured. As shown in Figure 6, 10% AGEs increased the intracellular ROS levels by approximately 50% compared to the control condition. Cortisol (3 nM) did not reduce the intracellular ROS levels, while quercetin alone did diminish the intracellular ROS levels. Figure 6 additionally shows that combining cortisol with quercetin led to a dose-dependent reduction of the intracellular ROS levels. Quercetin (5 µM) significantly reduced the intracellular ROS levels compared to 10% AGEs and 10% AGEs with cortisol.

## 4. Discussion

In the present study, it has been shown for the first time that dietary AGEs induced glucocorticoid resistance of macrophage-like (THP-1) cells. Exposure of THP-1 cells to dietary AGEs increased the production of IL8, indicating an inflammatory response, which was comparable to the pro-inflammatory effect of 3 ng/mL LPS. The glucocorticoid cortisol was able to decrease LPS-induced inflammation significantly better than AGE-induced inflammation. This indicates that exposure to dietary AGEs may lead to glucocorticoid resistance. Modulation of the pro-inflammatory response by different pharmacological compounds interfering in different steps of the anti-inflammatory mechanism of glucocorticoids showed that AGE-induced glucocorticoid resistance can be restored. The use of glucocorticoids is increasing due to the increased prevalence of chronic inflammatory diseases, such as asthma, rheumatoid arthritis, and IBD. Despite the effectiveness of glucocorticoids, some patients are resistant to these drugs. Glucocorticoid resistance is a disorder that is characterized by insensitivity to glucocorticoids [3]. Although much research has already focused on the mechanism of glucocorticoid resistance, it is still not completely understood why some patients are insensitive to glucocorticoids, while others are not. Several mechanisms have been suggested to be involved in the development of resistance toward glucocorticoids. The present research aimed to identify the mechanism of AGE-induced glucocorticoid resistance by exposing THP-1 cells to different compounds that interfere at different stages of the anti-inflammatory mechanism of glucocorticoids.

Barnes et al. published an excellent review about the mechanisms of glucocorticoid resistance (Figure 3) [3]. Phosphorylation of the GR was suggested as one important cause of such resistance. Phosphorylation of the GR at Ser226 and 211 inhibits the binding of the activated GR to GRE, resulting in a decreased expression of anti-inflammatory genes and consequently an increased expression of pro-inflammatory genes. One of the factors responsible for the phosphorylation of the GC is mTOR. Our results show that the inhibition of mTOR by rapamycin increased the sensitivity of THP-1 cells to cortisol. However, no differences were found in the protein levels of phosphorylated GC (at Ser226 and 211) between the control conditions and the AGE-treated cells. Even though rapamycin increased the sensitivity, these results suggest that phosphorylation of the GC at the two serine residues is not the main mechanism for AGE-induced glucocorticoid resistance. Another suggested mechanism can be mediated through oxidative stress. Oxidative stressors such as ROS are able to inactivate HDAC2 through the formation of peroxynitrite and the subsequent nitration of tyrosine residues of HDAC2 [16,22], thereby inducing glucocorticoid resistance [23,24]. HDAC2 normally ensures chromatin remodeling of pro-inflammatory genes; hence, inactivation results in the increased expression of pro-inflammatory genes, including IL8 [8]. It is therefore expected that antioxidants could ameliorate the sensitivity to glucocorticoids by increasing HDAC2 activity. In the present study, AGEs were found to increase the formation of ROS in THP-1 cells, which was significantly reduced by the antioxidant quercetin. Our results further show that quercetin in combination with cortisol decreased IL8 production, while quercetin alone did not have this effect. The inhibition of IL8 secretion by the combination of quercetin and cortisol indicate that the anti-inflammatory effect of quercetin is dependent on cortisol. This can be explained by the improvement of the cortisol response by quercetin, which is in line with previously reported data from Mitani et al. [25] that proved that quercetin was able to restore glucocorticoid resistance in U937 monocytes. Our results show that AGE-induced glucocorticoid resistance is likely to be caused by the formation of ROS. A plausible explanation for this effect is the damage of HDAC2 by ROS, rendering it inactive [15,26,27,28]. The strong antioxidant quercetin is able to improve glucocorticoid sensitivity by neutralizing ROS. The HDAC activator theophylline did not have any effect on AGE-induced inflammation and glucocorticoid resistance. This could be due to the fact that only very low concentrations of theophylline restore HDAC activity [29]. Secondly, theophylline might in fact not act on HDAC2, as was found by Ito et al. [30]. Theophylline seems to work at HDAC1 and HDAC3 specifically, which could explain why no effect was seen in our study. However, since HDAC2 activation was not investigated in this study, future research may focus on the involvement of HDAC2 in AGE-induced glucocorticoid resistance.

**Figure 7:**
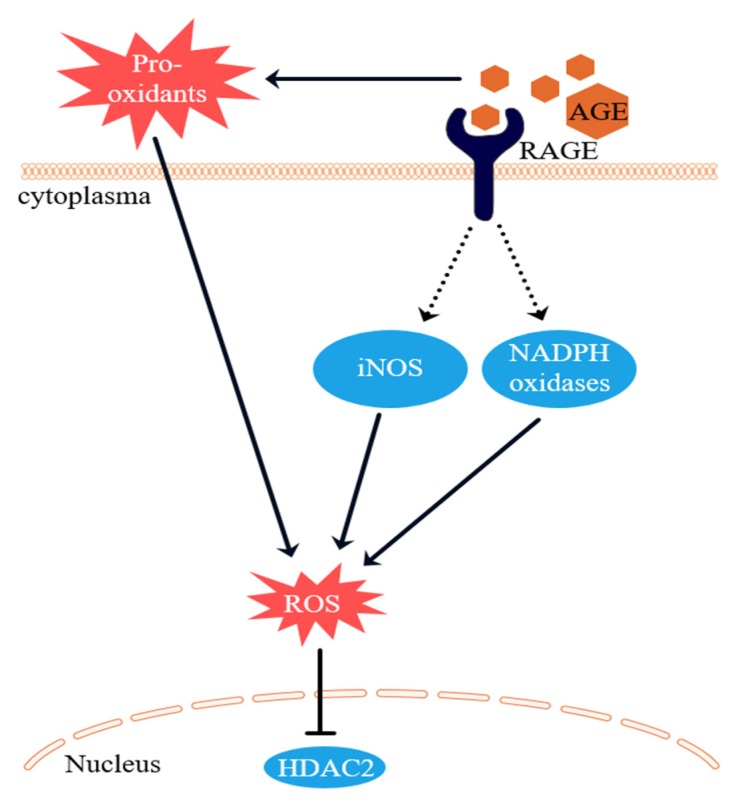
Proposed mechanism of glucocorticoid resistance caused by AGEs. The pro-oxidative AGEs lead to ROS formation. AGEs bind to RAGE. RAGE activates NADPH oxidases and upregulates iNOS which in turn induces ROS. Histone deacetylase 2 (HDAC2) is inhibited by ROS.

Based on the results of this study and existing literature, the proposed mechanism by which AGEs induce glucocorticoid resistance is shown in Figure 7. AGEs can induce ROS via two pathways. Firstly, AGEs bind to RAGE, which activates NADPH oxidases, leading to the formation of ROS. Activation of RAGE also eventually leads to an increased iNOS expression [22,31]. AGEs themselves are formed under oxidative conditions and are pro-oxidant molecules that are also able to form ROS [32,33]. This AGE-induced ROS formation then inactivates HDAC2, leading to glucocorticoid resistance. What is the implication of these findings for humans? It is under debate whether dietary AGEs are absorbed within the human body, but they are present within the gastrointestinal tract. Additionally, glucocorticoid resistance occurs in approximately 20% of people with IBD [1,2]. The results of the current study show that the type of food IBD patients eat may be of great importance to IBD patients suffering from glucocorticoid resistance. Studies are currently underway in our laboratory to analyze the digestion of dietary AGEs to investigate the effects of dietary AGEs on inflammation. In this way, we hope to get a better picture of the role of this study’s phenomena on the in vivo situation. Next to the potential negative effect of a high-AGE diet on glucocorticoid sensitivity, our study shows that at least one specific polyphenol, quercetin, can increase glucocorticoid responsiveness. Additionally, another polyphenol, curcumin, has been shown to have a similar effect in a COPD model [34]. People eating high-AGE diets, containing many fried and baked foods, might also eat less fruit and vegetables that contain many of the polyphenols in our diet. The question whether an inverse relation exists between AGE and polyphenol intake can be studied in the future.

## 5. Conclusions

In conclusion, the present study showed for the first time indications that inflammation induced by AGEs is less well mitigated by cortisol than LPS-induced inflammation. This indicates that AGEs may cause glucocorticoid resistance by increasing intracellular ROS. Quercetin is able to improve the glucocorticoid response by reducing the intracellular ROS levels.

## References

[1] Tung J., Loftus E.V., Freese D.K., El-Youssef M., Zinsmeister A.R., Melton L.J., Harmsen W.S., Sandborn W.J., Faubion W.A. (2006). A population-based study of the frequency of corticosteroid resistance and dependence in pediatric patients with Crohn’s disease and ulcerative colitis. Inflamm. Bowel Dis..

[2] Munkholm P., Langholz E., Davidsen M., Binder V. (1994). Frequency of glucocorticoid resistance and dependency in Crohn’s disease. Gut.

[3] Barnes P.J. (2010). Mechanisms and resistance in glucocorticoid control of inflammation. J. Steroid Biochem. Mol. Biol..

[4] Rhen T., Cidlowski J.A. (2005). Antiinflammatory action of glucocorticoids—New mechanisms for old drugs. N. Engl. J. Med..

[5] Ito K., Barnes P.J., Adcock I.M. (2000). Glucocorticoid receptor recruitment of histone deacetylase 2 inhibits interleukin-1beta-induced histone H4 acetylation on lysines 8 and 12. Mol. Cell. Biol..

[6] Clark A.R. (2003). MAP kinase phosphatase 1: A novel mediator of biological effects of glucocorticoids?. J. Endocrinol..

[7] Barnes P.J. (2006). Corticosteroid effects on cell signalling. Eur. Respir. J..

[8] Ito K., Yamamura S., Essilfie-Quaye S., Cosio B., Ito M., Barnes P.J., Adcock I.M. (2006). Histone deacetylase 2-mediated deacetylation of the glucocorticoid receptor enables NF-kappaB suppression. J. Exp. Med..

[9] Bergmann M.W., Staples K.J., Smith S.J., Barnes P.J., Newton R. (2004). Glucocorticoid inhibition of granulocyte macrophage-colony-stimulating factor from T cells is independent of control by nuclear factor-kappaB and conserved lymphokine element 0. Am. J. Respir. Cell Mol. Biol..

[10] Weigel N.L., Moore N.L. (2007). Steroid receptor phosphorylation: A key modulator of multiple receptor functions. Mol. Endocrinol..

[11] Matthews J.G., Ito K., Barnes P.J., Adcock I.M. (2004). Defective glucocorticoid receptor nuclear translocation and altered histone acetylation patterns in glucocorticoid-resistant patients. J. Allergy Clin. Immunol..

[12] Szatmary Z., Garabedian M.J., Vilcek J. (2004). Inhibition of glucocorticoid receptor-mediated transcriptional activation by p38 mitogen-activated protein (MAP) kinase. J. Biol. Chem..

[13] Miller A.L., Webb M.S., Copik A.J., Wang Y., Johnson B.H., Kumar R., Thompson E.B. (2005). p38 Mitogen-activated protein kinase (MAPK) is a key mediator in glucocorticoid-induced apoptosis of lymphoid cells: Correlation between p38 MAPK activation and site-specific phosphorylation of the human glucocorticoid receptor at serine 211. Mol. Endocrinol..

[14] Irusen E., Matthews J.G., Takahashi A., Barnes P.J., Chung K.F., Adcock I.M. (2002). p38 Mitogen-activated protein kinase-induced glucocorticoid receptor phosphorylation reduces its activity: Role in steroid-insensitive asthma. J. Allergy Clin. Immunol..

[15] Ito K., Hanazawa T., Tomita K., Barnes P.J., Adcock I.M. (2004). Oxidative stress reduces histone deacetylase 2 activity and enhances IL-8 gene expression: Role of tyrosine nitration. Biochem. Biophys. Res. Commun..

[16] Kirkham P., Rahman I. (2006). Oxidative stress in asthma and COPD: Antioxidants as a therapeutic strategy. Pharmacol. Ther..

[17] Ruijters E.J., Haenen G.R., Willemsen M., Weseler A.R., Bast A. (2016). Food-Derived Bioactives Can Protect the Anti-Inflammatory Activity of Cortisol with Antioxidant-Dependent and -Independent Mechanisms. Int. J. Mol. Sci..

[18] van der Lugt T., Weseler A., Gebbink W., Vrolijk M., Opperhuizen A., Bast A. (2018). Dietary Advanced Glycation Endproducts Induce an Inflammatory Response in Human Macrophages In Vitro. Nutrients.

[19] Xie J., Mendez J.D., Mendez-Valenzuela V., Aguilar-Hernandez M.M. (2013). Cellular signalling of the receptor for advanced glycation end products (RAGE). Cell. Signal..

[20] Poulsen M.W., Hedegaard R.V., Andersen J.M., de Courten B., Bugel S., Nielsen J., Skibsted L.H., Dragsted L.O. (2013). Advanced glycation endproducts in food and their effects on health. Food Chem. Toxicol..

[21] Ruiz-Leal M., George S. (2004). An In Vitro procedure for evaluation of early stage oxidative stress in an established fish cell line applied to investigation of PHAH and pesticide toxicity. Mar. Environ. Res..

[22] Bansal S., Siddarth M., Chawla D., Banerjee B.D., Madhu S.V., Tripathi A.K. (2012). Advanced glycation end products enhance reactive oxygen and nitrogen species generation in neutrophils In Vitro. Mol. Cell. Biochem..

[23] Barnes P.J., Adcock I.M. (2009). Glucocorticoid resistance in inflammatory diseases. Lancet.

[24] Ruijters E.J., Haenen G.R., Weseler A.R., Bast A. (2014). The cocoa flavanol (-)-epicatechin protects the cortisol response. Pharmacol. Res..

[25] Mitani A., Azam A., Vuppusetty C., Ito K., Mercado N., Barnes P.J. (2017). Quercetin restores corticosteroid sensitivity in cells from patients with chronic obstructive pulmonary disease. Exp. Lung Res..

[26] Barnes P.J. (2009). Role of HDAC2 in the pathophysiology of COPD. Annu. Rev. Physiol..

[27] To Y., Ito K., Kizawa Y., Failla M., Ito M., Kusama T., Elliott W.M., Hogg J.C., Adcock I.M., Barnes P.J. (2010). Targeting phosphoinositide-3-kinase-delta with theophylline reverses corticosteroid insensitivity in chronic obstructive pulmonary disease. Am. J. Respir. Crit. Care Med..

[28] Randall M.J., Haenen G.R., Bouwman F.G., van der Vliet A., Bast A. (2016). The tobacco smoke component acrolein induces glucocorticoid resistant gene expression via inhibition of histone deacetylase. Toxicol. Lett..

[29] Marwick J.A., Wallis G., Meja K., Kuster B., Bouwmeester T., Chakravarty P., Fletcher D., Whittaker P.A., Barnes P.J., Ito K. (2008). Oxidative stress modulates theophylline effects on steroid responsiveness. Biochem. Biophys. Res. Commun..

[30] Ito K., Lim S., Caramori G., Cosio B., Chung K.F., Adcock I.M., Barnes P.J. (2002). A molecular mechanism of action of theophylline: Induction of histone deacetylase activity to decrease inflammatory gene expression. Proc. Natl. Acad. Sci. USA.

[31] Wautier M.P., Chappey O., Corda S., Stern D.M., Schmidt A.M., Wautier J.L. (2001). Activation of NADPH oxidase by AGE links oxidant stress to altered gene expression via RAGE. Am. J. Physiol. Endocrinol. Metab..

[32] Rowan S., Bejarano E., Taylor A. (2018). Mechanistic targeting of advanced glycation end-products in age-related diseases. Biochim. Biophys. Acta Mol. Basis Dis..

[33] Nowotny K., Jung T., Hohn A., Weber D., Grune T. (2015). Advanced glycation end products and oxidative stress in type 2 diabetes mellitus. Biomolecules.

[34] Meja K.K., Rajendrasozhan S., Adenuga D., Biswas S.K., Sundar I.K., Spooner G., Marwick J.A., Chakravarty P., Fletcher D., Whittaker P. (2008). Curcumin restores corticosteroid function in monocytes exposed to oxidants by maintaining HDAC2. Am. J. Respir. Cell Mol. Biol..

